# Dynamin 1 Regulates Amyloid Generation through Modulation of BACE-1

**DOI:** 10.1371/journal.pone.0045033

**Published:** 2012-09-14

**Authors:** Li Zhu, Meng Su, Louise Lucast, Lijuan Liu, William J. Netzer, Samuel E. Gandy, Dongming Cai

**Affiliations:** 1 Department of Neurology and Alzheimer’s Disease Research Center, Mount Sinai School of Medicine, New York, New York, United States of America; 2 James J. Peters Veterans Affairs Medical Center, Bronx, New York, United States of America; 3 Department of Cell Biology and Program in Cellular Neuroscience, Neurodegeneration and Repair, Yale School of Medicine, New Haven, Connecticut, United States of America; 4 Laboratory of Molecular and Cellular Neuroscience, The Rockefeller University, New York, New York, United States of America; 5 Department of Pathology, John Hopkins Medical Center, Baltimore, Maryland, United States of America; Massachusetts General Hospital, United States of America

## Abstract

**Background:**

Several lines of investigation support the notion that endocytosis is crucial for Alzheimer’s disease (AD) pathogenesis. Substantial evidence have already been reported regarding the mechanisms underlying amyloid precursor protein (APP) traffic, but the regulation of beta-site APP-Cleaving Enzyme 1 (BACE-1) distribution among endosomes, TGN and plasma membrane remains unclear. Dynamin, an important adaptor protein that controls sorting of many molecules, has recently been associated with AD but its functions remain controversial. Here we studied possible roles for dynamin 1 (dyn1) in Aβ biogenesis.

**Principal Findings:**

We found that genetic perturbation of dyn1 reduces both secreted and intracellular Aβ levels in cell culture. There is a dramatic reduction in BACE-1 cleavage products of APP (sAPPβ and βCTF). Moreover, dyn1 knockdown (KD) leads to BACE-1 redistribution from the Golgi-TGN/endosome to the cell surface. There is an increase in the amount of surface holoAPP upon dyn1 KD, with resultant elevation of α–secretase cleavage products sAPPα and αCTF. But no changes are seen in the amount of nicastrin (NCT) or PS1 N-terminal fragment (NTF) at cell surface with dyn1 KD. Furthermore, treatment with a selective dynamin inhibitor Dynasore leads to similar reduction in βCTF and Aβ levels, comparable to changes with BACE inhibitor treatment. But combined inhibition of BACE-1 and dyn1 does not lead to further reduction in Aβ, suggesting that the Aβ-lowering effects of dynamin inhibition are mainly mediated through regulation of BACE-1 internalization. Aβ levels in dyn1^−/−^ primary neurons, as well as in 3-month old dyn1 haploinsufficient animals with AD transgenic background are consistently reduced when compared to their wildtype counterparts.

**Conclusions:**

In summary, these data suggest a previously unknown mechanism by which dyn1 affects amyloid generation through regulation of BACE-1 subcellular localization and therefore its enzymatic activities.

## Introduction

Late-onset Alzheimer’s disease (LOAD) typically manifests after the sixth decade, accounting for over 95% of all AD cases. Genetic studies of LOAD point to a number of risk factor genes, such as apolipoprotein E epsilon4 (ApoEε4) allele [1], and several endocytic proteins. For example, single nucleotide polymorphism (SNP) studies from two research groups studying Japanese and Belgian populations have independently identified an association of dynamin binding protein gene (DNMBP) on chromosome 10 to LOAD, particularly in individuals lacking the APOE ε4 allele [2], [3]. Furthermore, a significant association of LOAD with the dynamin 2 (DNM2) gene was detected by SNP analysis, especially in non-carriers of the ApoEε4 allele [4], [5].

Dynamin is a GTPase that plays a critical role in endocytic vesicle fission [6]. It is encoded by three different genes (DNM1, DNM2, and DNM3) in mammals [7]. Dynamin 1 (dyn1) is highly and selectively expressed in the nervous system and represents the major dynamin isoform expressed in this tissue [8]. Dyn1 has been linked to the biology of AD. For example, dominant-negative dyn1 (K44A mutant), which blocks endocytosis, reduces Aβ levels in interstitial spinal fluid (ISF) and prevents activity-dependent increases in Aβ [9]. Dyn1 K44A mutant also reduces oligomer Aβ_42_-induced neuronal death [10] and increases APP ectodomain shedding [11]. Others showed an increase in BACE-1 cleavage of APP and Aβ generation at the cell surface in dyn1 K44A expressing HeLa cells [12]. Taken together, a role for dyn1 in AD is implicated but precise molecular mechanism(s) remains elusive.

Herein, we report that using gene silencing techniques to knockdown dyn1 levels reduces both secreted and intracellular Aβ levels in cell culture. There is a dramatic reduction in beta-site APP-Cleaving Enzyme 1 (BACE-1) cleavage products of APP (sAPPβ and βCTF). Moreover, dyn1 knockdown (KD) leads to BACE-1 redistribution from the Golgi-TGN/endosome to the cell surface. There is an increase in the amount of surface holoAPP upon dyn1 KD, with resultant elevation of α–secretase cleavage products sAPPα and αCTF. But no changes are seen in the amount of nicastrin (NCT) or PS1 N-terminal fragment (NTF) at cell surface with dyn1 KD. Furthermore, treatment with a selective dynamin inhibitor Dynasore leads to similar reduction in βCTF and Aβ levels, comparable to changes with BACE inhibitor treatment. But combined inhibition of BACE-1 and dyn1 does not lead to further reduction in Aβ, suggesting that the Aβ-lowering effects of dynamin inhibition are mainly mediated through regulation of BACE internalization. Aβ Levels in dyn1^−/−^ primary neurons, as well as in 3-month old dyn1 haploinsufficient animals with AD transgenic background are consistently reduced when compared to their wildtype counterparts.

In summary, these data suggest a previously unknown modulatory mechanism by which dyn1 affects amyloid generation through regulation of BACE-1 subcellular localization and therefore its enzymatic activities. Together, our findings provide mechanistic evidence that inhibition of dyn1 functions may prevent certain pathologic changes associated with AD.

## Materials and Methods

### Ethics Statements

All animal studies were carried out in strict accordance with the recommendations in the Guide for the Care and Use of Laboratory Animals of the National Institutes of Health. The animal studies were approved by the Institutional Animal Care and Use Committee of James J Peters VA Medical Center (Permit Number: CAI-10-044a). All efforts were made to minimize suffering.

### Antibodies

4G8, 6E10 and sAPPβ (Covance), anti-APP N-terminus MAB348 (clone 22C11, Millipore) and anti-BACE C-terminus MAB5308 (clone 61, Millipore), anti-dynamin clone 41, anti-EEA1, anti-γ-adaptin and anti-nicastrin (NCT) (BD Transduction), anti-β actin (Santa Cruz), anti-Pen-2 antibody NE1008 (Calbiochem), anti-mouse and rabbit HRP conjugates (Vector Laboratories), Texas- Red conjugated anti-mouse IgG (Vector Laboratories Inc.) were purchased. pAb369 (C-terminal APP antibody) [13], RU690 (C-terminal BACE1 antibody) [14], RUWN-b1 (C-terminal sAPPβ antibody), RU717 (N-terminal nicastrin antibody) [15] and Ab14 (N-terminal PS1 antibody) [16] generously provided by Dr. Greengard at The Rockefeller University, were also used for APP/CTFs, BACE1, sAPPβ, NCT and PS1 detection. Synthetic sAPPβ peptide (Covance) and BACE-1 lysates from 293 cells (Santa Cruz Biotechnology) were used as positive controls.

### Cell Lines

N2a cells stably transfected with cDNAs encoding human APP695 provided by Dr. Greengard at The Rockefeller University [17] were transfected with dyn1siRNA and maintained for 4–5 days to achieve about 50–80% knockdown of dyn1 protein levels. Fibroblast cell lines from dynamin 1, dynamin 2 conditional knockout mice expressing tamoxifen-inducible Cre recombinase (dyn KD) provided by Dr. Pietro De Camilli at Yale School of Medicine [18] lose dynamin expression upon treatment with 1µM 4-hydroxy-tamoxifen. Wild type fibroblast cells were treated by tamoxifen as a control. Alternatively, cells were treated with a dyn inhibitor Dynasore [19] at 10 µM (Sigma), or a BACE inhibitor at 15 µM (Inhibitor IV, EMD) overnight before subjected for further analysis.

### Neuronal Cultures

Postnatal day 0–1 cortical neurons from dyn1^+/+^ or dyn1^−/−^ mouse pups [8] were cultured on poly-D-lysine (Sigma) pre-coated dishes for 14–21 days.

### siRNA Oligonucleotides

The siRNA duplexes specifically targeting dyn1 (IDT Inc.) were synthesized. Sequences are as follows: forward 5′-rGrGrC rUrUrA rCrArU rGrArA rUrArC rCrArA rCrCrA rCrGA A-3′; reverse, 5′-rUrUrC rGrUrG rGrUrU rGrGrU rArUrU rCrArU rGrUrA rArGrCrCrArG-3′. Stealth siRNA Control GC Duplex was used as a negative control (IDT Inc.). Sequences are as follows: forward 5′-rCrGrU rUrArA rUrCrG rCrGrU rArUrA rCrGrC rGrUA T-3′; reverse, 5′-rArUrA rCrGrC rGrUrA rUrUrA rUrArC rGrCrG rArArC rGrArC-3′.

### Cell Transfection

For siRNA analysis, N2a 695 cells were seeded at 50% to 60% confluence and transfected with 200 pmol siRNA dyn1 versus control duplex using Lipofectamine RNAimax (Invitrogen). For transient transfection of cDNA constructs, dyn KD fibroblasts were seeded at 70% confluence and transfected with 0.5 µg of BACE1-EGFP cDNA provided by Dr. Bradley Hyman at Harvard Medical Center, [20] using Lipofectamine 2000 (Invitrogen).

### Cell Lysate Analysis

After transfection, cells were harvested in lysis buffer [21]. Equal amounts of total protein were loaded for Western blot using 6E10 to detect holoAPP (full-length APP). Aβ_40_ and Aβ_42_ levels in media were determined by human ELISA kits (WAKO), according to the manufacturer’s instructions. Alternatively, mouse Aβ_40_ levels in media of Dyn KD fibroblasts after various treatments were determined by mouse ELISA kits (WAKO).

### Immunoprecipitation

Media or lysates were diluted with immunoprecipitation buffer [22] and immunoprecipitated using 4G8 followed by immunoblotted with 6E10 for Aβ and βCTF detection. Media were immunoprecipitated using MAB348 (Millipore) followed by immunoblotted with sAPPβ antibody (for sAPPβ) or 6E10 antibody (for sAPPα).

### Biotinylation/Cell Surface Assay

Dyn KD fibroblasts were incubated at 4°C with 0.5 mg/ml sulfo N-hydroxysuccinimide biotin (Pierce) to label surface proteins as described previously [17]. Samples of both biotinylated and non-biotinylated BACE-1 were analyzed by Western blot using MAB5308 or RU690. Purified lysates from BACE-1 expressing 293 cells were used as positive controls. Alternatively, samples of both biotinylated and non-biotinylated holoAPP were analyzed by Western blot using 6E10.

### Immunofluorescence Confocal Microscopy

Dyn KD fibroblasts transfected with EGFP-BACE1 were treated with or without tamoxifen for dyn1 knockdown, followed by fixation and permeabilization. Cells were then incubated with primary antibody against an endosomal marker EEA1 (1∶1000) or a Golgi/TGN marker γ-adaptin (1∶1000) following incubation with Texas Red conjugated anti-mouse IgG (1∶1000). Immunofluorescence staining was examined by confocal microscopy (LSM510, Zeiss).

### Measurement of Newly Synthesized Aβ Production

Primary neurons derived from dyn1^+/+^ or dyn1^−/−^ animals [8] were labeled with [^35^S] methionine (500 µCi/ml) for 5 hours at 37°C. Media was immunoprecipitated with 4G8 and then subjected to autoradiography to determine levels of Aβ_1–40/42_ and Aβ_11–40/42_ [23], [24].

### Generation of Dyn1 Haploinsufficient Mice with AD TRANSGENIC Mouse Background

Human Swedish βAPP and FAD-linked PS1 ΔE9 mutant transgenic mice from Jackson Laboratory [25], [26] were mated with heterozygous dyn1 null mice (dyn1^+/−^) provided by Dr. Pietro De Camilli from Yale School of Medicine [8]. Double heterozygous F1s were then bred with heterozygous dyn1 null mice only to generate offsprings that express human Swedish APP and FAD-linked PS1 ΔE9 in the dyn1^+/+^ or dyn1^+/−^ background. Genotypes were determined by PCR amplification as described previously.

### Brain Lysate Preparation and Analysis

Mouse brains from APP/PS1^+/−^ dyn1^+/+^ or APP/PS1^+/−^ dyn1^+/−^ at 2–3 months of age, were rapidly dissected and snap frozen for further analysis. Each frozen hemi-brain was then processed via step-wise solubilization [21], [27]. Lysates were analyzed by Western blot using 6E10 to determine levels of holoAPP and βCTF. Levels of Aβ_40_ were determined by human Aβ_40_ ELISA kits (WAKO), according to the manufacturer’s instructions. Results were normalized to wet brain weight and expressed as picomoles per gram.

### Statistical Analysis

Densitometric analysis of western blot bands (integrated density) was performed using Multigauge v3.1 software (Fujifilm). Levels of holoAPP, βCTF, sAPPβ/α, Aβ and BACE-1 were normalized to actin and expressed as percentage of control. Absolute Aβ_40_ concentrations were quantitatively determined by sandwich ELISA (Wako). Independent-samples *t* tests (parametric design) were used to determine significant mean differences between groups. Significance for *t* tests are reported with a *p<*0.05 using two-tailed tests with an α level of 0.05. All statistical analysis was performed using SPSS v18.0.

## Results

### 1. Dynamin 1 (Dyn1) Knockdown in N2a 695 Cells Reduces Aβ and βCTF Levels

**Figure 1:**
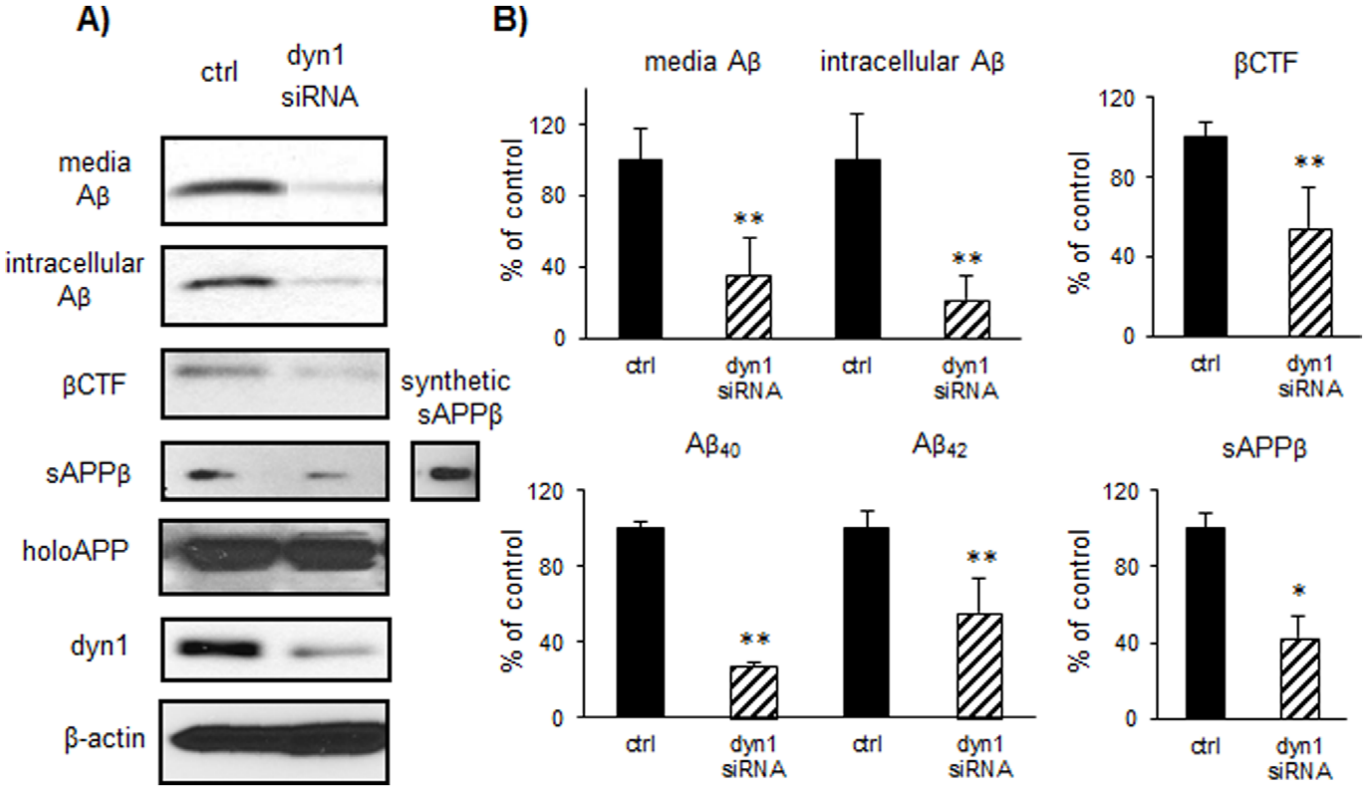
Dynamin 1 (dyn1) knockdown of in N2a 695 cells reduces Aβ and βCTF levels. A) N2a 695 cells were treated with dyn1 or control siRNA for analysis of APP metabolism. Levels of media and intracellular Aβ, sAPPβ, βCTF and holoAPP were determined by immunoprecipitation with 4G8 or MAB348 followed by immunoblotting with 6E10 or sAPPβ antibody. Recombinant sAPPβ peptide was used as a positive control. Levels of β-actin were also determined. B) Protein levels were normalized to β-actin and expressed as percentage of control. Data were collected in duplicate or triplicate from three independent experiments. Significant reductions (**p<0.001) in Aβ, βCTF and sAPPβ (*p<0.05) were observed upon dyn1 knockdown, as compared to control. Levels of Aβ_40_ and Aβ_42_ in the media are determined by sandwich ELISA analysis (**p<0.001).

To determine whether dyn1 modulates APP metabolism, we first analyzed the effects of dyn1 knockdown on levels of APP and its metabolites in N2a cells stably expressing human APP695 (N2a 695 cells). Using siRNA transfection methods, 50–80% reduction in dyn1 protein levels was achieved after 4–5 days. Both secreted and intracellular Aβ levels were significantly decreased by 64.5% and 77.8% respectively, compared to control siRNA transfection (Fig.1A, *p*<0.001). There was 62.5% reduction in Aβ_40_ and 44.7% reduction in Aβ_42_ levels measured by sandwich ELISA (Fig.1B, *p*<0.001). There was a dramatic reduction in other APP metabolites sAPPβ and βCTF levels (57.4%, *p* = 0.021 and 45.7%, *p*<0.001 respectively), without significant changes in holoAPP levels (119.16% of controls, *p* = 0.101). In addition, the levels of BACE-1 as well as γ-secretase components such as PS1 NTF and Pen-2 were unchanged (Figure S1A).

To confirm the specificity of effects of dyn1 siRNA on APP metabolism, three different dynamin 1 siRNA duplex constructs were synthesized (IDT Inc.) and transfected into N2a 695 cells to determine APP processing. Secreted Aβ levels were similarly decreased by all three siRNA constructs compared to control siRNA transfection (Figure S1B). The specificity of Aβ detection was determined by comparing to transfection of APP siRNA. Moreover, to confirm the specificity of βCTF detection, lysates from N2a 695 cells with dyn1 or control siRNA transfection were immunoprecipitated with 4G8 followed by immunoblotting with 6E10 (Figure S1B, left panels) to determine levels of βCTF and Aβ. Alternatively, lysates were directly blotted with 6E10 to detect holoAPP, βCTF and Aβ, followed by reprobing with 369 for detection of α/βCTF (Figure S1C, right panels).

Based on our findings, the consistent changes in levels of βCTF and sAPPβ support the notion that dyn1 knockdown inhibits APP cleavage by BACE-1 rather than accelerating degradation of βCTF.

### 2. Dyn1 Down-regulation Changes BACE-1 Intracellular Distribution

Next, we evaluated the effects of dyn1 knockdown on BACE1 intracellular traffic using dyn1 knockdown (KD) fibroblasts derived from dyn conditional knockout mice expressing tamoxifen-inducible Cre recombinase [18]. These experimental conditions allowed us to study dynamic changes in BACE-1 with inducible dyn1 down-regulation. After tamoxifen treatment at 1µM for 5–7 days, dyn1 protein levels were reduced by 56.6% (Figure S2A, *p* = 0.03) in the KD fibroblasts. Alternatively, wildtype fibroblast cells were treated with tamoxifen as a control and there was no change in dyn1 protein levels with tamoxifen treatment (Figure S2B, right panel). Similar to the results using dyn1 siRNA in the N2a 695 cell line, reduced βCTF levels (Figure S2A; 66.7% of reduction, *p* = 0.008) were observed in dyn1 KD fibroblasts with tamoxifen treatment. Tamoxifen treatment in wildtype cells did not cause any changes in α/βCTF levels, ruling out an effect of tamoxifen itself (Figure S2B, left panel).

**Figure 2:**
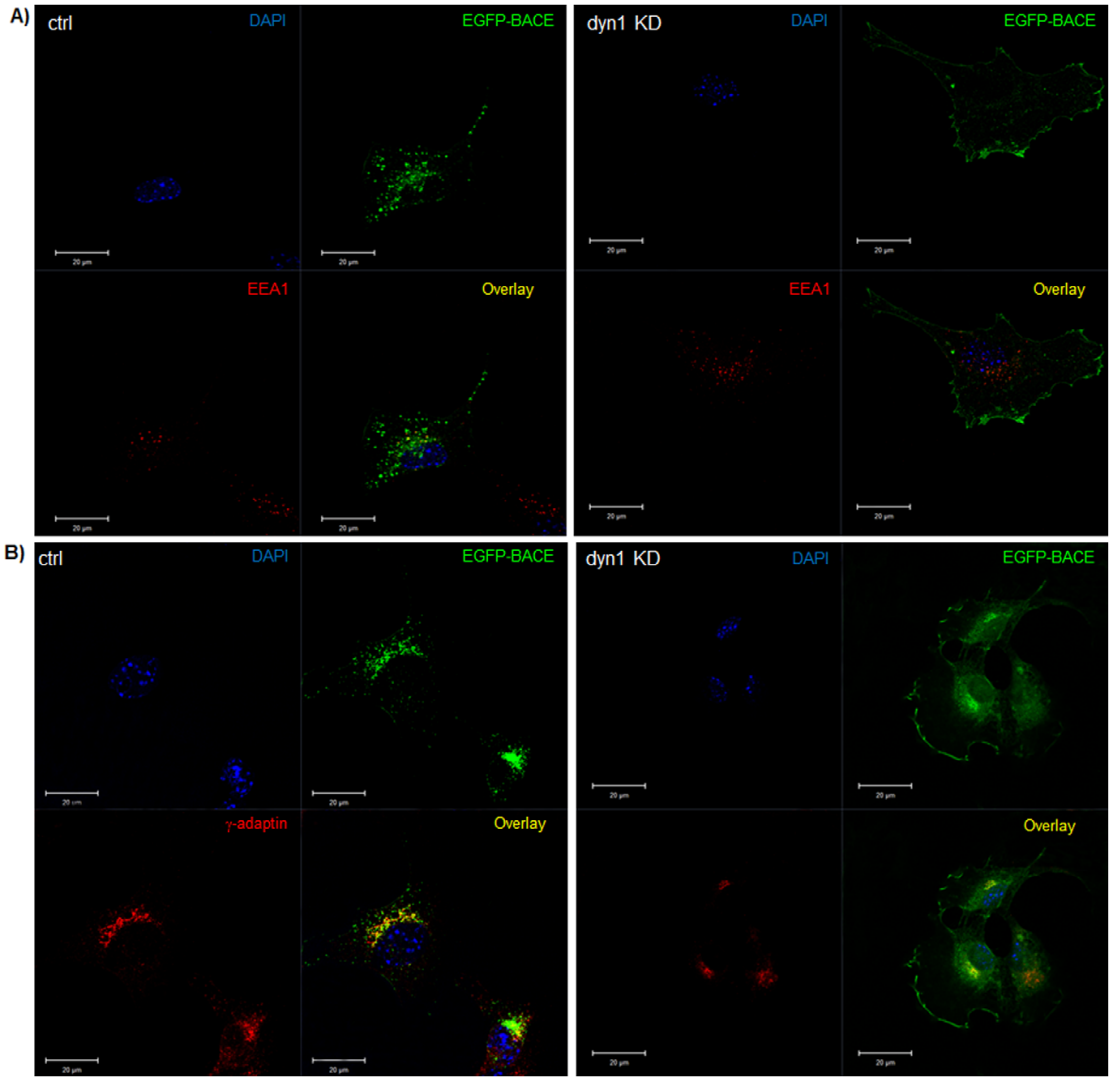
Dyn1 down-regulation changes BACE-1 intracellular distribution. A) Dyn1 fibroblast cells treated without (control; ctrl) or with tamoxifen (dyn1 KD) were transiently transfected with EGFP-BACE-1 followed by fixation. Localization of BACE-1 is shown as green fluorescence (labeled as EGFP-BACE). In addition, cells were double stained with an endosomal marker, EEA1 (red; labeled as EEA1). Overlay of both signals is shown as yellow (labeled as Overlay). Nuclear staining by DAPI is shown as blue (labeled as DAPI). B) Localization of BACE-1 is shown as green fluorescence (labeled as EGFP-BACE). In addition, cells were double stained with a TGN marker, γ-adaptin (red; labeled as γ-adaptin). Overlay of both signals is shown as yellow (labeled as Overlay). Nuclear staining by DAPI is shown as blue (labeled as DAPI). BACE-1 (green fluorescence) is mainly localized in the endosomes and TGN/Golgi in control cells. However, after dyn1 knockdown, the amounts of green fluorescence (EGFP-BACE1) are dramatically increased at cell surface with a little remaining within the TGN/Golgi and endosomes.

BACE-1 intracellular distribution in dyn1 KD cells was studied after transfection with EGFP-BACE1 construct [20]. As shown in Fig. 2, amounts of EGFP-BACE-1 at the cell surface were significantly increased with dyn1 knockdown. Under control conditions, BACE-1 (green fluorescence) was mainly localized in the endosomes and TGN/Golgi, determined by an endosomal marker EEA1 (red fluorescence, Fig. 2A,) as well as a TGN marker γ-adaptin (red fluorescence, Figure 2B). However, after dyn1 knockdown, amounts of green fluorescence (EGFP-BACE1) were dramatically increased at cell surface with little remaining within the TGN/Golgi and endosomes. Transfected EGFP-BACE-1 expression levels were comparable between control cells and dyn1 KD cells (Figure S3A).

### 3. Dyn1 Knockdown Increases Cell Surface BACE-1 and holoAPP Levels

**Figure 3:**
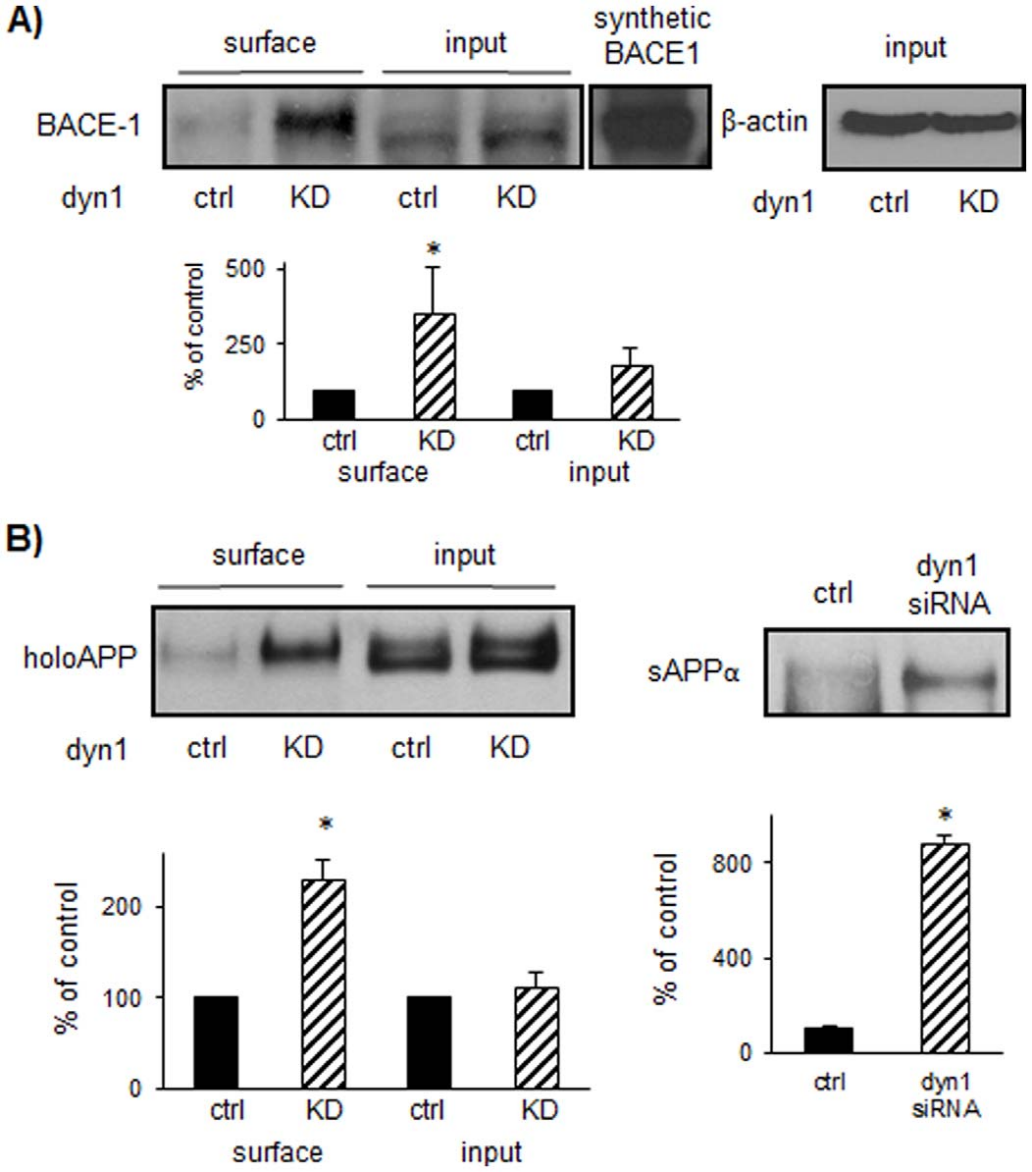
Dyn1 knockdown increases cell surface BACE-1 and holoAPP levels. A) Dyn KD fibroblast cells were treated with tamoxifen to induce dyn1 knockdown and then labeled with biotin followed by immunoprecipitation with streptavidin beads to pull down surface BACE-1. The amount of endogenous BACE-1 at cell surface was compared between control and dyn1 KD conditions. Total protein levels of endogenous BACE-1 were also determined from lysates as input. Protein levels were normalized to β-actin content and expressed as percentage of control. Data were collected in duplicate or triplicate from three independent experiments. Elevations in BACE-1 at cell surface (*p<0.05) without significant changes in total protein levels, were observed upon dyn1 KD. Cell lysates of BACE-1 expressing 293 cells were used as positive controls in these experiments. B) Alternatively, the amount of surface holoAPP was compared between control and DKO conditions. Elevations in holoAPP at cell surface (*p<0.05) without significant changes in total protein levels, were observed upon dyn1 KD. The sAPPα levels were determined in N2a 695 cells after dyn1 siRNA transfection, by immunoprecipitation with MAB348 followed by immunoblotting with 6E10 antibody. The levels of sAPPα was increased by 286.3% (*p* = 0.042) upon dyn1 KD.

Using surface biotinylation methods, we next analyzed the intracellular and surface distribution of endogenous BACE-1 in dyn1 KD fibroblasts without over-expression of EGFP-BACE-1. As shown in Fig.3A (top panel), surface BACE-1 levels were elevated by 251.8% with dyn1 knockdown when compared to controls (*p* = 0.047). There was a modest elevation in total BACE-1 protein levels without achieving statistical significance (increase by 78.5%, *p* = 0.08). The specificity of BACE-1 detection was confirmed by using cell lysates extracted from BACE-1 expressing 293 cells as positive controls.

Interestingly, cell surface holoAPP levels in dyn KD cells were also elevated by 131.1% (*p* = 0.02) upon tamoxifen induction, without any significant changes in total APP protein levels (111.6% of controls, *p* = 0.615) as shown in Figure 3B. In addition, the levels of sAPPα in N2a 695 cells determined by immunoprecipitation with MAB348 followed by immunoblotting with 6E10 antibody, was increased by 286.3% (*p* = 0.042) upon dyn1 knockdown by siRNA transfection. Consistently, the levels of αCTF were also elevated with dyn1 KD in both N2a 695 cells (Figure S1C) and in dyn1 KD fibroblasts (Figure S2A; increase by 84.9%, *p* = 0.011). The increased holoAPP at cell surface likely promotes α-secretase cleavage to generate sAPPα and αCTF.

However, it should be noted that dyn1 knockdown does not have a general effect on internalization of all proteins because no significant changes were seen in the amounts of nicastrin (NCT) or PS1 NTF at cell surface upon dyn KD conditions (Figure S3B). In addition, the total protein levels of NCT and NCT were unchanged with dyn1 KD (Figure S3B, input panels). The changes in both BACE-1 and holoAPP at cell surface with dyn1 KD suggest that dyn1 regulates a common trafficking pathway for those two proteins between endosomes and plasma membrane.

Taken together, these data suggest that dyn1 knockdown inhibits endocytosis of BACE-1, thereby preventing delivery of BACE-1 to its active sites in endosomes [28]. The BACE-1 on the cell surface is likely to be inactive and unable to process holoAPP to generate βCTF and Aβ. Therefore, increased holoAPP availability at surface for α-secretase cleavage leads to elevation in sAPPα (Figure 3B) and αCTF (Figure S1C and S2A).

### 4. A Selective Dynamin Inhibitor Dynasore Reduces βCTF and Aβ Levels

**Figure 4:**
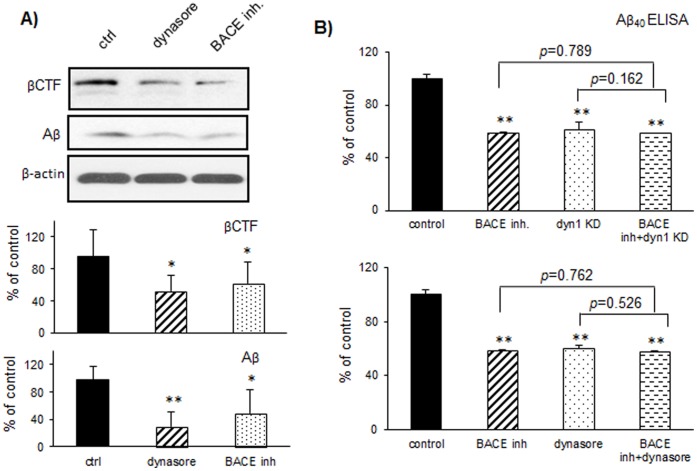
A selective dynamin inhibitor Dynasore reduces βCTF and Aβ levels. A) N2a 695 cells were treated with dynasore at 10 µM or BACE Inhibitor IV at 15µM before subjected for further analysis. Levels of βCTF and Aβ were determined and significant reductions were observed upon dynasore or BACE inhibitor treatment, as compared to control (*p<0.05; **, *p*<0.001). Protein levels were normalized to β-actin and expressed as percentage of control. Data were collected in duplicate or triplicate from three independent experiments. B) Dyn1 KD fibroblast cells were treated with tamoxifen (to induce dyn1 knockdown), BACE inhibitor IV or combination of both (top panels). Levels of Aβ_40_ in the media are determined by sandwich ELISA analysis (**p<0.001 comparing each treatment or combination to control). Alternatively cells were treated with dynasore, or BACE inhibitor IV or combination of both (bottom panels). Levels of Aβ_40_ in the media are determined by sandwich ELISA analysis (**p<0.001 comparing each treatment or combination to control).

Next, to determine whether pharmacological inhibition of dyn1 could affect APP processing, N2a 695 cells were treated with a selective dynamin inhibitor dynasore at 10µM [19], [29], [30]. As shown in Figure 4A, levels of βCTF and Aβ secreted in media were decreased by 47.4% (*p* = 0.008) and 70.4% (*p*<0.001), comparable to that with a BACE inhibitor treatment [31] (reduction of βCTF by 38% *p* = 0.006, and Aβ by 50.4% *p* = 0.011).

Furthermore, the effects of dyn1 knockdown on BACE-1 cleavage of endogenous APP were confirmed in dyn1 KD fibroblast cell lines. Similar to the results using siRNA and chemical inhibitors in N2a 695 cells, reduction in βCTF levels (Figure S2A; 66.7% of reduction, *p* = 0.008) was observed in these cells upon dyn1 knockdown. The total Aβ_40_ levels measured by sandwich ELISA were reduced by 39.4% (*p<*0.001), comparable to the reduction induced by a BACE inhibitor treatment (41.3% reduction, *p<*0.001; Fig. 4B). However, the combination of BACE inhibitor IV with tamoxifen-induced dyn1 KD did not lead to further reduction in Aβ_40_ levels than either treatment alone (41.3% reduction). Consistently, Aβ_40_ levels were reduced by 40% in dyn1 KD fibroblasts with dynasore treatment alone. The combination of dynasore and BACE inhibitor treatment did not lead to further reduction in Aβ_40_ levels as well (43.5% reduction). The lack of additive effects with combined treatments suggests that the Aβ-lowering effects of dyn1 KD, dynasore and BACE inhibitor IV all act through the same step: BACE1 inhibition.

### 5. Genetic Perturbation of Dyn1 in Animals Reduces Aβ Generation

**Figure 5:**
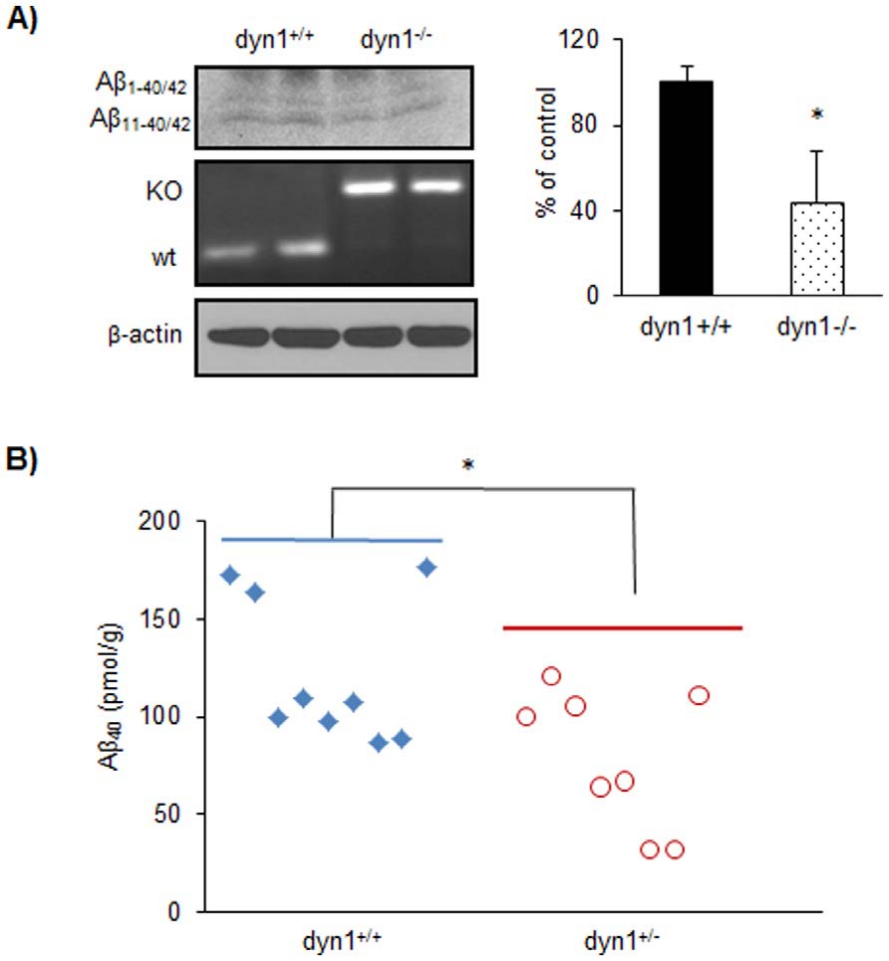
Genetic perturbation of dyn1 in animals reduces Aβ generation. A) Cultured cortical neurons derived from dyn1^+/+^ and dyn1^−/−^ animals were labeled with S^35^-methionine for 5 hours. The levels of S^35^-labeled Aβ (both Aβ_1–40/42_ and Aβ_11–40/42_ species) were determined and reduction in both Aβ species (*p<0.05) was observed in dyn1^−/−^ neurons. The dyn1 genotypes were determined by PCR (middle panel, KO band ∼217 bp and wt band ∼146 bp). The levels of β-actin were also determined in dyn1^+/+^ and dyn1^−/−^ primary neuron lysates. Data were collected in duplicate or triplicate from three independent experiments. B) Levels of Aβ_40_ in hemi-brain lysates of 3-month old APP/PS1^+/−^ dyn1^+/+^ (n = 9) and APP/PS1^+/−^ dyn1^+/−^ (n = 8) mice were determined by sandwich ELISA analysis. Statistically significant reduction in Aβ_40_ was observed in APP/PS1^+/−^ dyn1^+/−^ mice in comparison to their control littermates (APP/PS1^+/−^ dyn1^+/+^; *p<0.05). Data were presented as pmol/gram of wet brain weight of each animal.

Dyn1 has been implicated in regulating activity-dependent Aβ secretion through endocytosis [9]. To determine whether dyn1 has a direct effect on endogenous Aβ generation, we next analyzed levels of newly synthesized Aβ secreted from dyn1^−/−^ neurons [8] using S^35^-methionine labeling approach. When compared to dyn1^+/+^ neurons, levels of S^35^-labeled Aβ (both Aβ_1–40/42_ and Aβ_11–40/42_ species as described previously [23], [24]) secreted into the media were decreased by 55.9% in dyn1^−/−^ neurons after 5 hours of incubation (Fig. 5A, *p* = 0.004). The dyn1 genotypes were confirmed by PCR results (Fig. 5A, middle panel) and the levels of β-actin were comparable between dyn1^+/+^ and dyn1^−/−^ neurons (Fig.5A, bottom panel).

Dyn1 knockout animals can only survive up to 2 weeks [8]. To determine whether genetic perturbation of dyn1 affects Aβ generation in adult animals *in vivo*, we next analyzed the levels of Aβ, holoAPP and βCTF in the brains of dyn1 haploinsufficient (dyn1^+/−^) mice with AD transgenic background expressing human Swedish APP and PS1ΔE9 mutations [25], [26]. At 2–3 months of age, levels of Aβ_40_ determined by sandwich ELISA in total brain lysates from AD transgenic mice carrying dyn1 haploinsufficiency (n = 8), were decreased by 35.5% when compared to controls (n = 9; *p* = 0.013; Fig. 5B). However, levels of Aβ_42_ at this age group were too low for detection of any statistically significant changes. The levels of βCTF in the brains of dyn1 haploinsufficient animals (dyn1^+/−^) were slightly decreased (13.7%) when compared to dyn1 wild-type (dyn1^+/+^) animals (Figure S4). There was a trend of reduction in βCTF in dyn1^+/−^ mouse brains, but due to large variations in these transgenic animals (even within the same group), this effect did not achieve statistical significance (*p* = 0.613). The levels of holoAPP were comparable between the two groups (Figure S4∶107.4% of controls in dyn1^+/−^ mice; *p* = 0.679).

## Discussion

It is well established that sorting mechanisms which cause APP, BACE-1 and γ-secretase to colocalize in the same subcellular compartment play important roles in the regulation of Aβ production [32]. Further, endocytic pathways are important for regulation of BACE-1 activity (Huse et al., 2000). Substantial data regarding the nature and mechanisms underlying APP endocytosis have been reported, but the role of endocytosis in regulating BACE-1 distribution among endosomes, TGN and plasma membrane remain unclear.

As an important endocytic adaptor protein, the involvement of dynamin in Aβ biogenesis has been described [9], [11], [12], raising the possibility that it may affect Aβ biogenesis as a step in the pathogenesis of AD. However, there are discrepancies between prior reports regarding its effects on Aβ. One group showed increased cell surface Aβ production with dominant negative dyn1 K44A [12] but other groups reported reduced Aβ production by dyn1 K44A [9], [11]. It was also suggested that impaired APP endocytosis by dyn1 K44A contributes to reduction in activity-dependent Aβ secretion [9]. A similar situation has been proposed for deficiencies of other sorting proteins (e.g., SorL1 and Vps35), both of which, like dynamin, will undoubtedly alter sorting of multiple substrates. Yet, deficiencies of either SorL1 or Vps35 can promote Aβ generation and accumulation as prominent phenotypes at the whole brain level [21], [33].

Our data, using gene silencing in three different cell lines (including N2a cells overexpressing human APP695 cells, fibroblasts with endogenous APP expression, and primary neurons derived from dyn1^−/−^ animals), as well as in animal models, demonstrates a previously unknown mechanism by which dyn1 regulates Aβ generation; i.e., through modulation of BACE-1 traffic between the plasma membrane and endosomes. By reducing BACE-1 localization to the endosomal compartment, dyn1 inhibits Aβ generation and secretion. These data further support the idea that endosomes are the most optimal compartments for BACE-1 enzymatic activities and that prevention of BACE-1 distribution into endosomes can reduce APP processing into Aβ. The reduction of both Aβ and βCTF/sAPPβ through dyn1 knockdown may be a better therapeutic strategy compared to those targeting γ-secretase, given that recent data suggest β- but not γ-secretase-mediated proteolysis of APP causes synaptic and memory deficits in AD [34].

We would suggest that the previously reported effects of dyn1 knockdown on Aβ generation [9], [11], [12] are more likely to be attributable to modulation of BACE-1 sorting and less likely to be attributable to the conventional wisdom that dyn1 is primarily acting via reduction of APP internalization. A combination of dynasore (or dyn1 knockdown) and BACE inhibitor treatment did not cause any further reduction in levels of Aβ generation when compared with the maximal effect of either treatment on its own (Figure 4B), consistent with the interpretation that they both act on the same pathway. If dynamin knockdown/blockade caused an inhibition of Aβ generation by acting via inhibition of APP internalization, then there would be further reduction of Aβ when the BACE inhibitor was added. Together, our data suggest that the Aβ-lowering effects of dynamin inhibition are mainly mediated through regulation of BACE-1 internalization.

Interestingly, a recent report demonstrated that an immediate early gene, Arc/Arg3.1 which recruits dynamin and endophilin to early/recycling endosomes at post-synaptic terminals, can mediate PS1/γ-secretase trafficking through the endosomal pathway, and thereby affect activity-dependent Aβ generation [35]. These data suggest that dyn1-regulated endocytosis may also regulate γ-secretase function. However, our data demonstrate that dyn1 knockdown affects only BACE-1 and holoAPP traffic to plasma membrane but not the γ-secretase components nicastrin and PS1 NTF (Figure S3B), suggesting a shared pathway for BACE-1/holoAPP selectively regulated by dyn1.

### Conclusions

In summary, here we report a previously unknown regulatory mechanism by which dyn1 modulates APP proteolysis and Aβ homeostasis through regulation of BACE-1 subcellular distribution. Without directly targeting BACE-1 enzymatic activities, modulation of BACE-1 subcellular localization by targeting dyn1 suggests new directions for developing therapeutic strategies for AD.

## Supporting Information

Figure S1 **Genetic silencing of dyn1 in N2a cells reduces βCTF and Aβ levels.** A) Western blot analysis of PS1 NTF by Ab14, Pen-2 by rabbit polyclonal anti Pen-2 antibody NE1008 (Calbiochem), and BACE-1 by clone 61 and RU690, were performed in N2a695 cells with transfection of dyn1 siRNA or control duplex. There were no significant differences in total protein levels of BACE-1 or γ-secretase components with dyn1 KD conditions. B) Three different dyn1 siRNA duplex were synthesized (IDT Inc.) and transfected into N2a 695 cells to determine the specificity of observed effects of dyn1 KD on APP processing. The secreted Aβ levels were similarly decreased in three different dyn1 siRNA treatment, compared to control siRNA transfection. The specificity of Aβ detection was determined by comparing to transfection of APP siRNA. Sequences of the dyn1siRNAs are as follows: Duplex 1 forward 5′-rGrGrC rUrUrA rCrArU rGrArA rUrArC rCrArA rCrCrA rCrGA A-3′; reverse, 5′-rUrUrC rGrUrG rGrUrU rGrGrU rArUrU rCrArU rGrUrA rArGrCrCrArG-3′. Duplex 2 forward 5′-rGrGrA rCrArU rArGrA rCrGrG rCrArA rGrArCrATC-3′; reverse, 5′-rGrArU rGrUrC rCrUrU rCrUrU rGrCrC rGrUrC rUrArU rGrUrC rCrUrU-3′. Duplex 3 forward 5′-rCrGrG rUrUrA rGrArC rArGrU rGrCrA rCrCrA rArGrA rArGrC T-3′; reverse, 5′-rArGrC rUrUrC rUrUrG rGrUrG rCrArC rUrGrU rCrUrA rArCrC rGrUrG-3′. Stealth siRNA Control Medium GC Duplex was used as a negative control (IDT Inc.). Sequences are described as before. APP siRNA sequences are as follows: forward 5′-rUrCrC rUrCrC rGrUrC rUrUrG rArUrA rUrUrU rGrUrC rArArC rCrCrA-3′, reverse 5′-rGrGrU rUrGrA rCrArA rArUrA rUrCrA rArGrA rCrGrg rArGGA-3′. C) Media and lysates from N2a 695 cells with dyn1 or control siRNA transfection were immunoprecipitated with 4G8 followed by immunoblotted with 6E10 to determine levels of βCTF and Aβ. Alternatively, lysates were directly blotted with 6E10 to detect holoAPP, βCTF and Aβ, followed by reprobed with 369 for detection of α/βCTF.
(TIF)

Figure S2 **Dyn1 knockdown in fibroblast cells changes endogenous APP processing.** A) Dyn1 KD cells were treated with tamoxifen to induce dyn1 knockdown. After tamoxifen treatment at 1 µM for 5–7 days, dyn1 protein levels were reduced by 56.6% (*p* = 0.03) in the dyn1 KD cells. Similar to the results in N2a 695 cells, changes in metabolism of endogenous APP including reduction in βCTF levels (66.7% of reduction; *p* = 0.008) were observed in these cells upon dyn1 knockdown. Elevation of αCTF levels was also observed (84.9% of increase; *p* = 0.011). B) Wild type fibroblast cells were treated with tamoxifen at 1 µM for 5–7 days as a control. The levels of βCTF and αCTF, as well as dyn1 protein levels were unchanged with or without tamoxifen treatment (ctrl versus Tamoxifen).
(TIF)

Figure S3 **Dyn1 knockdown increases cell surface BACE-1 levels.** A) Dyn KD fibroblast cells were treated with tamoxifen to induce dyn1 knockdown followed by transiently transfected with EGFP-BACE-1. The levels of EGFP-BACE1 in dyn DKO cells with or without tamoxifen induction (three independent transfection experiments in both control and DKO cells) were comparable as shown in top panel (determined by Invitrogen anti-GFP antibody 3E6). The levels of dyn1 and actin were also determined (bottom panels). B) The amounts of nicastrin (NCT) or PS1 NTF at cell surface were also determined by biotinylation followed by streptavidin pull down. Western blot analysis of surface and total (input) NCT by anti-nicastrin antibody (BD Transduction) and RU717, as well as surface and total (input) PS1 NTF by Ab14 was performed. No significant changes were seen in the amounts of NCT and PS1NTF at cell surface or total cell lysates upon dyn KD conditions.
(TIF)

Figure S4 **Genetic perturbation of dyn1** **in animals slightly reduces βCTF levels.** Levels of βCTF and holoAPP in total hemi-brain lysates of 3-month old APP/PS1^+/−^ dyn1^+/+^ (n = 9) and APP/PS1^+/−^ dyn1^+/−^ (n = 8) mice were determined by western blot with antibody 6E10. Levels of βCTF and holoAPP were normalized to actin and presented as ratio to wild type counterparts. The levels of βCTF in the brains of dyn1 haploinsufficient animals were slightly decreased (13.7%) when compared to dyn1 wild-type animals. But due to large variations in these transgenic animals, this effect did not achieve statistical significance (*p* = 0.613). The levels of holoAPP were comparable between two groups (107.4% of controls in dyn1^+/−^ mice; *p* = 0.679).
(TIF)

## References

[1] Waring SC, Rosenberg RN (2008) Genome-wide association studies in Alzheimer disease. Arch Neurol 65: 329–334. 18332245 10.1001/archneur.65.3.329

[2] Bettens K, Brouwers N, Engelborghs S, De Pooter T, De Deyn PP, et al (2009) DNMBP is genetically associated with Alzheimer dementia in the Belgian population. Neurobiol Aging 30: 2000–2009. 18359537 10.1016/j.neurobiolaging.2008.02.003

[3] Kuwano R, Miyashita A, Arai H, Asada T, Imagawa M, et al (2006) Dynamin-binding protein gene on chromosome 10q is associated with late-onset Alzheimer's disease. Hum Mol Genet 15: 2170–2182. 16740596 10.1093/hmg/ddl142

[4] Kamagata E, Kudo T, Kimura R, Tanimukai H, Morihara T, et al (2009) Decrease of dynamin 2 levels in late-onset Alzheimer's disease alters Abeta metabolism. Biochem Biophys Res Commun 379: 691–695. 19126407 10.1016/j.bbrc.2008.12.147

[5] Aidaralieva NJ, Kamino K, Kimura R, Yamamoto M, Morihara T, et al (2008) Dynamin 2 gene is a novel susceptibility gene for late-onset Alzheimer disease in non-APOE-epsilon4 carriers. J Hum Genet 53: 296–302. 18236001 10.1007/s10038-008-0251-9

[6] Roux A, Uyhazi K, Frost A, De Camilli P (2006) GTP-dependent twisting of dynamin implicates constriction and tension in membrane fission. Nature 441: 528–531. 16648839 10.1038/nature04718

[7] Cao H, Garcia F, McNiven MA (1998) Differential distribution of dynamin isoforms in mammalian cells. Mol Biol Cell 9: 2595–2609. 9725914 10.1091/mbc.9.9.2595 PMC25532

[8] Ferguson SM, Brasnjo G, Hayashi M, Wolfel M, Collesi C, et al (2007) A selective activity-dependent requirement for dynamin 1 in synaptic vesicle endocytosis. Science 316: 570–574. 17463283 10.1126/science.1140621

[9] Cirrito JR, Kang JE, Lee J, Stewart FR, Verges DK, et al (2008) Endocytosis is required for synaptic activity-dependent release of amyloid-beta in vivo. Neuron 58: 42–51. 18400162 10.1016/j.neuron.2008.02.003 PMC2390913

[10] Yu C, Nwabuisi-Heath E, Laxton K, Ladu MJ (2010) Endocytic pathways mediating oligomeric Abeta42 neurotoxicity. Mol Neurodegener 5: 19. 20478062 10.1186/1750-1326-5-19 PMC2881055

[11] Carey RM, Balcz BA, Lopez-Coviella I, Slack BE (2005) Inhibition of dynamin-dependent endocytosis increases shedding of the amyloid precursor protein ectodomain and reduces generation of amyloid beta protein. BMC Cell Biol 6: 30. 16095541 10.1186/1471-2121-6-30 PMC1208872

[12] Chyung JH, Selkoe DJ (2003) Inhibition of receptor-mediated endocytosis demonstrates generation of amyloid beta-protein at the cell surface. J Biol Chem 278: 51035–51043. 14525989 10.1074/jbc.M304989200

[13] Buxbaum JD, Gandy SE, Cicchetti P, Ehrlich ME, Czernik AJ, et al (1990) Processing of Alzheimer beta/A4 amyloid precursor protein: modulation by agents that regulate protein phosphorylation. Proc Natl Acad Sci U S A 87: 6003–6006. 2116015 10.1073/pnas.87.15.6003 PMC54458

[14] Yan R, Han P, Miao H, Greengard P, Xu H (2001) The transmembrane domain of the Alzheimer's beta-secretase (BACE1) determines its late Golgi localization and access to beta -amyloid precursor protein (APP) substrate. J Biol Chem 276: 36788–36796. 11466313 10.1074/jbc.M104350200

[15] Leem JY, Vijayan S, Han P, Cai D, Machura M, et al (2002) Presenilin 1 is required for maturation and cell surface accumulation of nicastrin. J Biol Chem 277: 19236–19240. 11943765 10.1074/jbc.C200148200

[16] Levitan D, Lee J, Song L, Manning R, Wong G, et al (2001) PS1 N- and C-terminal fragments form a complex that functions in APP processing and Notch signaling. Proc Natl Acad Sci U S A 98: 12186–12190. 11593035 10.1073/pnas.211321898 PMC59789

[17] Cai D, Leem JY, Greenfield JP, Wang P, Kim BS, et al (2003) Presenilin-1 regulates intracellular trafficking and cell surface delivery of beta-amyloid precursor protein. J Biol Chem 278: 3446–3454. 12435726 10.1074/jbc.M209065200

[18] Ferguson SM, Raimondi A, Paradise S, Shen H, Mesaki K, et al (2009) Coordinated actions of actin and BAR proteins upstream of dynamin at endocytic clathrin-coated pits. Dev Cell 17: 811–822. 20059951 10.1016/j.devcel.2009.11.005 PMC2861561

[19] Macia E, Ehrlich M, Massol R, Boucrot E, Brunner C, et al (2006) Dynasore, a cell-permeable inhibitor of dynamin. Dev Cell 10: 839–850. 16740485 10.1016/j.devcel.2006.04.002

[20] von Arnim CA, Tangredi MM, Peltan ID, Lee BM, Irizarry MC, et al (2004) Demonstration of BACE (beta-secretase) phosphorylation and its interaction with GGA1 in cells by fluorescence-lifetime imaging microscopy. J Cell Sci 117: 5437–5445. 15466887 10.1242/jcs.01422

[21] Lane RF, Raines SM, Steele JW, Ehrlich ME, Lah JA, et al (2010) Diabetes-associated SorCS1 regulates Alzheimer's amyloid-beta metabolism: evidence for involvement of SorL1 and the retromer complex. J Neurosci 30: 13110–13115. 20881129 10.1523/JNEUROSCI.3872-10.2010 PMC3274732

[22] Cai D, Netzer WJ, Zhong M, Lin Y, Du G, et al (2006) Presenilin-1 uses phospholipase D1 as a negative regulator of beta-amyloid formation. Proc Natl Acad Sci U S A 103: 1941–1946. 16449386 10.1073/pnas.0510708103 PMC1413665

[23] Netzer WJ, Dou F, Cai D, Veach D, Jean S, et al (2003) Gleevec inhibits beta-amyloid production but not Notch cleavage. Proc Natl Acad Sci U S A 100: 12444–12449. 14523244 10.1073/pnas.1534745100 PMC218777

[24] Xu H, Gouras GK, Greenfield JP, Vincent B, Naslund J, et al (1998) Estrogen reduces neuronal generation of Alzheimer beta-amyloid peptides. Nat Med 4: 447–451. 9546791 10.1038/nm0498-447

[25] Jankowsky JL, Fadale DJ, Anderson J, Xu GM, Gonzales V, et al (2004) Mutant presenilins specifically elevate the levels of the 42 residue beta-amyloid peptide in vivo: evidence for augmentation of a 42-specific gamma secretase. Hum Mol Genet 13: 159–170. 14645205 10.1093/hmg/ddh019

[26] Jankowsky JL, Slunt HH, Ratovitski T, Jenkins NA, Copeland NG, et al (2001) Co-expression of multiple transgenes in mouse CNS: a comparison of strategies. Biomol Eng 17: 157–165. 11337275 10.1016/s1389-0344(01)00067-3

[27] Kawarabayashi T, Younkin LH, Saido TC, Shoji M, Ashe KH, et al (2001) Age-dependent changes in brain, CSF, and plasma amyloid (beta) protein in the Tg2576 transgenic mouse model of Alzheimer's disease. J Neurosci 21: 372–381. 11160418 10.1523/JNEUROSCI.21-02-00372.2001 PMC6763819

[28] Huse JT, Pijak DS, Leslie GJ, Lee VM, Doms RW (2000) Maturation and endosomal targeting of beta-site amyloid precursor protein-cleaving enzyme. The Alzheimer's disease beta-secretase. J Biol Chem 275: 33729–33737. 10924510 10.1074/jbc.M004175200

[29] Chung C, Barylko B, Leitz J, Liu X, Kavalali ET (2010) Acute dynamin inhibition dissects synaptic vesicle recycling pathways that drive spontaneous and evoked neurotransmission. J Neurosci 30: 1363–1376. 20107062 10.1523/JNEUROSCI.3427-09.2010 PMC2823378

[30] Kirchhausen T, Macia E, Pelish HE (2008) Use of dynasore, the small molecule inhibitor of dynamin, in the regulation of endocytosis. Methods Enzymol 438: 77–93. 18413242 10.1016/S0076-6879(07)38006-3 PMC2796620

[31] Stachel SJ, Coburn CA, Steele TG, Jones KG, Loutzenhiser EF, et al (2004) Structure-based design of potent and selective cell-permeable inhibitors of human beta-secretase (BACE-1). J Med Chem 47: 6447–6450. 15588077 10.1021/jm049379g

[32] Small SA, Gandy S (2006) Sorting through the cell biology of Alzheimer's disease: intracellular pathways to pathogenesis. Neuron 52: 15–31. 17015224 10.1016/j.neuron.2006.09.001 PMC4820242

[33] Wen L, Tang FL, Hong Y, Luo SW, Wang CL, et al (2011) VPS35 haploinsufficiency increases Alzheimer's disease neuropathology. J Cell Biol 195: 765–779. 22105352 10.1083/jcb.201105109 PMC3257571

[34] Tamayev R, Matsuda S, Arancio O, D'Adamio L (2011) beta- but not gamma-secretase proteolysis of APP causes synaptic and memory deficits in a mouse model of dementia. EMBO Mol Med. 10.1002/emmm.201100195 PMC3376850 22170863

[35] Wu J, Petralia RS, Kurushima H, Patel H, Jung MY, et al (2011) Arc/Arg3.1 Regulates an Endosomal Pathway Essential for Activity-Dependent beta-Amyloid Generation. Cell 147: 615–628. 22036569 10.1016/j.cell.2011.09.036 PMC3207263

