# Supplementary figures and images for: Dynamin 1 Regulates Amyloid Generation through Modulation of BACE-1
Source: PLoS One. 2012 Sep 14;7(9):e45033. doi: 10.1371/journal.pone.0045033 (PMC3443198; doi:10.1371/journal.pone.0045033)

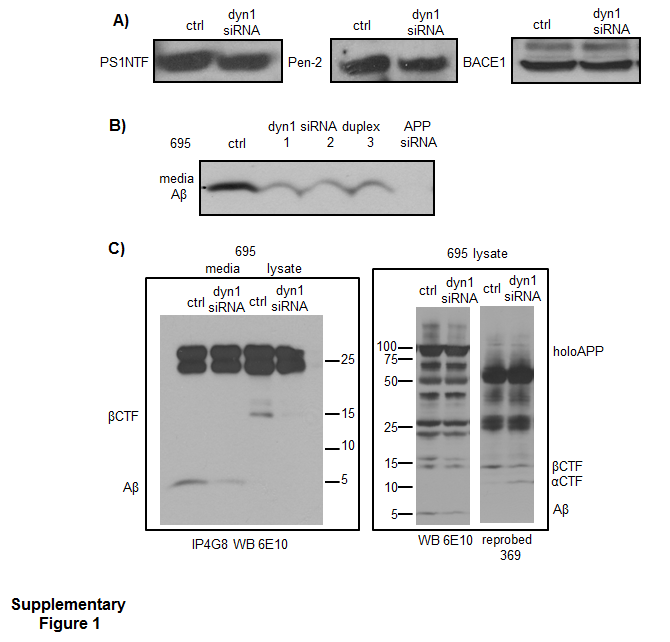

Supplement: Figure S1 — Genetic silencing of dyn1 in N2a cells reduces βCTF and Aβ levels. A) Western blot analysis of PS1 NTF by Ab14, Pen-2 by rabbit polyclonal anti Pen-2 antibody NE1008 (Calbiochem), and BACE-1 by clone 61 and RU690, were performed in N2a695 cells with transfection of dyn1 siRNA or control duplex. There were no significant differences in total protein levels of BACE-1 or γ-secretase components with dyn1 KD conditions. B) Three different dyn1 siRNA duplex were synthesized (IDT Inc.) and transfected into N2a 695 cells to determine the specificity of observed effects of dyn1 KD on APP processing. The secreted Aβ levels were similarly decreased in three different dyn1 siRNA treatment, compared to control siRNA transfection. The specificity of Aβ detection was determined by comparing to transfection of APP siRNA. Sequences of the dyn1siRNAs are as follows: Duplex 1 forward 5′-rGrGrC rUrUrA rCrArU rGrArA rUrArC rCrArA rCrCrA rCrGA A-3′; reverse, 5′-rUrUrC rGrUrG rGrUrU rGrGrU rArUrU rCrArU rGrUrA rArGrCrCrArG-3′. Duplex 2 forward 5′-rGrGrA rCrArU rArGrA rCrGrG rCrArA rGrArCrATC-3′; reverse, 5′-rGrArU rGrUrC rCrUrU rCrUrU rGrCrC rGrUrC rUrArU rGrUrC rCrUrU-3′. Duplex 3 forward 5′-rCrGrG rUrUrA rGrArC rArGrU rGrCrA rCrCrA rArGrA rArGrC T-3′; reverse, 5′-rArGrC rUrUrC rUrUrG rGrUrG rCrArC rUrGrU rCrUrA rArCrC rGrUrG-3′. Stealth siRNA Control Medium GC Duplex was used as a negative control (IDT Inc.). Sequences are described as before. APP siRNA sequences are as follows: forward 5′-rUrCrC rUrCrC rGrUrC rUrUrG rArUrA rUrUrU rGrUrC rArArC rCrCrA-3′, reverse 5′-rGrGrU rUrGrA rCrArA rArUrA rUrCrA rArGrA rCrGrg rArGGA-3′. C) Media and lysates from N2a 695 cells with dyn1 or control siRNA transfection were immunoprecipitated with 4G8 followed by immunoblotted with 6E10 to determine levels of βCTF and Aβ. Alternatively, lysates were directly blotted with 6E10 to detect holoAPP, βCTF and Aβ, followed by reprobed with 369 for detection of α/βCTF. (TIF) [file pone.0045033.s001.tif]

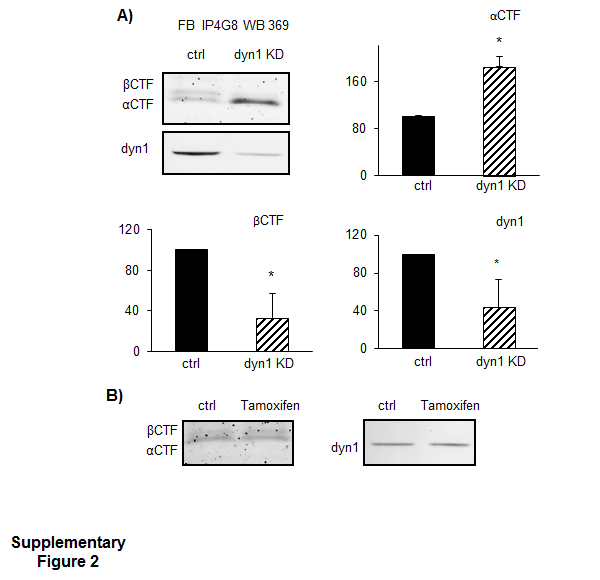

Supplement: Figure S2 — Dyn1 knockdown in fibroblast cells changes endogenous APP processing. A) Dyn1 KD cells were treated with tamoxifen to induce dyn1 knockdown. After tamoxifen treatment at 1 µM for 5–7 days, dyn1 protein levels were reduced by 56.6% (p = 0.03) in the dyn1 KD cells. Similar to the results in N2a 695 cells, changes in metabolism of endogenous APP including reduction in βCTF levels (66.7% of reduction; p = 0.008) were observed in these cells upon dyn1 knockdown. Elevation of αCTF levels was also observed (84.9% of increase; p = 0.011). B) Wild type fibroblast cells were treated with tamoxifen at 1 µM for 5–7 days as a control. The levels of βCTF and αCTF, as well as dyn1 protein levels were unchanged with or without tamoxifen treatment (ctrl versus Tamoxifen). (TIF) [file pone.0045033.s002.tif]

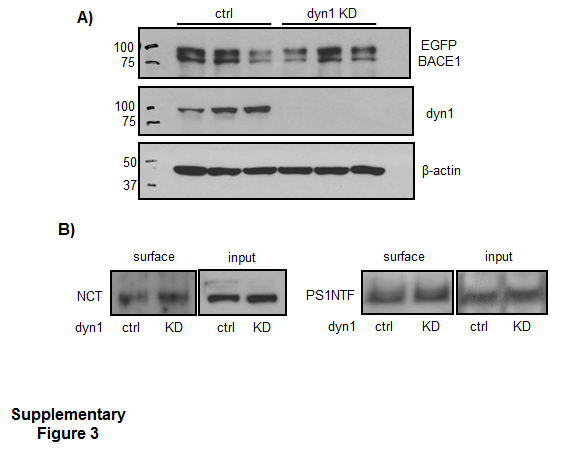

Supplement: Figure S3 — Dyn1 knockdown increases cell surface BACE-1 levels. A) Dyn KD fibroblast cells were treated with tamoxifen to induce dyn1 knockdown followed by transiently transfected with EGFP-BACE-1. The levels of EGFP-BACE1 in dyn DKO cells with or without tamoxifen induction (three independent transfection experiments in both control and DKO cells) were comparable as shown in top panel (determined by Invitrogen anti-GFP antibody 3E6). The levels of dyn1 and actin were also determined (bottom panels). B) The amounts of nicastrin (NCT) or PS1 NTF at cell surface were also determined by biotinylation followed by streptavidin pull down. Western blot analysis of surface and total (input) NCT by anti-nicastrin antibody (BD Transduction) and RU717, as well as surface and total (input) PS1 NTF by Ab14 was performed. No significant changes were seen in the amounts of NCT and PS1NTF at cell surface or total cell lysates upon dyn KD conditions. (TIF) [file pone.0045033.s003.tif]

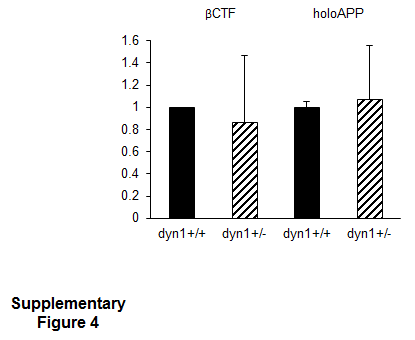

Supplement: Figure S4 — Genetic perturbation of dyn1 in animals slightly reduces βCTF levels. Levels of βCTF and holoAPP in total hemi-brain lysates of 3-month old APP/PS1+/− dyn1+/+ (n = 9) and APP/PS1+/− dyn1+/− (n = 8) mice were determined by western blot with antibody 6E10. Levels of βCTF and holoAPP were normalized to actin and presented as ratio to wild type counterparts. The levels of βCTF in the brains of dyn1 haploinsufficient animals were slightly decreased (13.7%) when compared to dyn1 wild-type animals. But due to large variations in these transgenic animals, this effect did not achieve statistical significance (p = 0.613). The levels of holoAPP were comparable between two groups (107.4% of controls in dyn1+/− mice; p = 0.679). (TIF) [file pone.0045033.s004.tif]
